# Fetal Growth Trajectories and Measures of Insulin Resistance in Young Adults

**DOI:** 10.1210/clinem/dgad292

**Published:** 2023-01-29

**Authors:** Ashish Yadav, Lawrence J Beilin, Rae-Chi Huang, John P Newnham, Scott W White, Trevor A Mori

**Affiliations:** UWA Medical School, University of Western Australia, Perth, 6009 WA, Australia; UWA Medical School, University of Western Australia, Perth, 6009 WA, Australia; Nutrition Health Innovation Research Institute, Edith Cowan University, Perth, 6027 WA, Australia; Division of Obstetrics and Gynaecology, University of Western Australia, Perth, 6009 WA, Australia; Division of Obstetrics and Gynaecology, University of Western Australia, Perth, 6009 WA, Australia; UWA Medical School, University of Western Australia, Perth, 6009 WA, Australia

**Keywords:** fetal growth, trajectories, insulin resistance, diabetes, cardiometabolic risk

## Abstract

**Context:**

Events during gestation greatly influence the risk of cardiometabolic diseases including diabetes in offspring during later life.

**Objective:**

This study aimed to investigate relationships between serial ultrasound-derived fetal growth trajectories and markers of insulin resistance in young adults in the Raine Study, an Australian pregnancy cohort.

**Methods:**

Linear mixed modeling examined the relationship between fetal growth trajectory groups, constructed using serial ultrasound-based abdominal circumference (AC), femur length (FL), and head circumference (HC) from 1333 mother-fetal pairs, and offspring Homeostatic Model Assessment of Insulin Resistance (HOMA-IR), as a marker of diabetes risk, at 20 (n = 414), 22 (n = 385), and 27 (n = 431) years. Analyses were adjusted for age, sex, ethnicity, socioeconomic status, adult lifestyle factors, and maternal factors during pregnancy.

**Results:**

The study identified 7 AC, 5 FL, and 5 HC growth trajectory groups. Compared to the average-stable (reference) group, a low-falling AC growth trajectory (26%; *P* = .005) and 2 low HC growth trajectories (20%; *P* = .006% and 8%; *P* = .021) were associated with higher adult HOMA-IR. Trajectories representing a high-stable FL and a rising HC were associated with 12% (*P* = .002) and 9% (*P* = .021) lower adult HOMA-IR, respectively, compared to the reference group.

**Conclusion:**

Restricted fetal HC and AC from early pregnancy are associated with higher relative insulin resistance in the offspring during adulthood. These data strengthen our understanding of the importance of the intrauterine environment and its effect on the risk of predisposition to adult diabetes and related metabolic disorders.

Diabetes has emerged as a serious, life-threatening, and disabling chronic disease with more than 1 in 10 adults presently living with diabetes worldwide (1). In 2021, 536.6 million people, accounting for approximately 10.5% of the world's adult population, had either type 1 or type 2 diabetes, which is projected to rise to 12.2% (783.2 million) in 2045 (1). An estimated 1.3 million Australians (4.9%) had diabetes in 2020, including 48 300 people newly diagnosed with type 2 diabetes mellitus (T2DM) in the year 2020 (2). T2DM is associated with a 2 to 3 times higher risk of premature death compared to the general population (3, 4). The rising prevalence over the past 2 decades has led to growing concerns and prompted research into the developmental and environmental factors that contribute to the onset and progression of T2DM (5-8). Sedentary lifestyle, consumption of energy-dense foods, and increase in overweight and obesity are associated with a greater risk of early-onset T2DM (4, 9).

There is considerable evidence that events during gestation may predispose to increased risk of adult cardiometabolic diseases, including diabetes (10-12). Birth weight has been extensively studied as a surrogate marker of fetal growth; low birth weight is closely associated with adult diabetes (5-7, 13). However, birthweight does not necessarily provide an accurate measure of early fetal growth and development. In the Raine Study, an Australian longitudinal pregnancy cohort, we have shown a disassociation between birth weight and growth trajectories in utero (14). We have also reported significant associations between different intrauterine growth trajectories and offspring adult blood pressure (14), body-mass index (BMI), waist circumference, and high-sensitivity C-reactive protein (hs-CRP) (15).

The present study extends our recent findings and investigates the relationships between fetal growth trajectories and adult Homeostatic Model Assessment of Insulin Resistance (HOMA-IR) as a marker of insulin resistance.

## Materials and Methods

The Raine Study enrolled 2900 pregnant women from 1989 to 1991 in Perth, Western Australia. The study aimed to investigate the effect of ultrasound imaging on pregnancy outcomes and the role of early-life events on subsequent childhood and adult health. Details on recruitment, randomization, and data collection in the original trial have been published previously (16). In brief, the study randomly assigned pregnant women (Gen1) into 2 groups based on the number of ultrasounds administered during gestation. While the intervention arm had 5 imaging examinations at 18, 24, 28, 34, and 38 weeks’ gestation, the control arm had a single ultrasound at 18 weeks unless clinically required. Only participants from the intervention arm were selected for the present analysis as a minimum of 2 ultrasound measurements were needed to develop growth trajectories. Offspring (Gen2) (n = 2868) have been prospectively followed up from birth to their current age of 27 years. Demographic, lifestyle, clinical, and biochemical information has been collected at regular intervals through questionnaires and clinical assessments. The present analysis uses Gen2 cohort information at 20, 22, and 27-years. Informed, written consent was provided by the pregnant women during initial recruitment and the adult offspring at each follow-up. The Human Research Ethics Committees at King Edward Memorial Hospital and The University of Western Australia approved the study.

### Gen1 (Pregnancy) Demographic and Lifestyle Measures

Information on Gen1 maternal and paternal sociodemographic characteristics and ethnicity, mother's marital status, family income, maternal smoking, and alcohol drinking was obtained by self-reported questionnaires at 16 and 34 weeks’ gestation. Annual family income during pregnancy was used to assess family income, low being less than $24 000 (AUS) during 1989 to 1991. Maternal medical records provided pregnancy characteristics including maternal weight, height, and medical conditions. Gestational age was calculated from the date of the last menstrual period and in case of discordancy, ultrasound estimation at 18 weeks. Maternal BMI was calculated at 16 weeks, and pregnancy weight gain was calculated between 16 and 34 weeks’ gestation. Gestational weight gain during this period is associated with in utero growth and birth weight. Smoking and alcohol drinking during pregnancy were assessed at 16 and 34 weeks and recorded as yes/no, yes being consumption at either or both time points. Self-reported gestational diabetes was recorded 2 days after delivery. All live births at less than 37 completed weeks were categorized as preterm. Standard blood pressure recordings during pregnancy obtained by midwives were used to establish pregnancy hypertension (HTN), characterized as uncomplicated HTN or complicated HTN. History of HTN before pregnancy or HTN during pregnancy without proteinuria or preterm delivery was defined as uncomplicated HTN. Complicated HTN was defined as HTN during pregnancy with proteinuria (>2 + on dipstix test) or 300 mg on 24-hour urinary protein excretion or preterm delivery (17). Systolic blood pressure greater than 140 mm Hg and/or diastolic blood pressure greater than 90 mm Hg was classified as HTN during pregnancy (18). Birth weight of the offspring was extracted from hospital records. Breast feeding was coded as not breastfed, breastfed for less than 6 months, or breastfed for 6 months or longer.

### Adult Offspring (Gen2) Measures

Wedderburn Chair Scales (to the nearest 100 g) were used to measure body weight with participants dressed in light clothing. A Stadiometer (to the nearest 0.1 cm) recorded height while a measuring tape was used to measure waist circumference (to the nearest 0.1 cm). A halfway point between the lowest rib and the iliac crest was used to record waist circumference. Sociodemographic and lifestyle data at 20, 22, and 27 years were obtained from computer-based questionnaires. Smoking was coded as a binary variable (yes/no) with participants categorized as smokers if they smoked a cigarette in the past 1 month. Total ethanol consumption in grams per week was obtained for alcohol intake, with 1 standard drink equivalent to 10-g ethanol. Information included the amount and type of alcoholic beverage consumed daily over the past 7 days. In females, current use of any hormonal contraceptive pill, injection, implants, or intrauterine device determined the status of hormonal contraceptives (yes/no). Offspring were categorized as White if both parents were White. Socioeconomic indexes for areas (SEIFA) scores were used as a continuous variable to quantify the socioeconomic status (SES) of Gen2 participants. Education was categorized as completing high school (level 1), apprenticeship or vocational training (level 2), or university (level 3). Metabolic equivalents (MET-min/wk) were used to measure physical activity; one MET was defined as the amount of oxygen consumed during rest (3.5 mL/kg/min). MET-minutes per week were obtained using the International Physical Activity Questionnaire (IPAQ-short/long) as per a standardized protocol (19). BMI was coded as a continuous variable (body weight in kg/height in m^2^). Fasting plasma glucose (mmol/L) was assayed by standard spectrophotometer (Abbott Diagnostics, Abbott Laboratories. Serum insulin was measured by the immunoassay technique (Abbott Diagnostics, Abbott Laboratories). HOMA-IR was calculated according to the formula: [fasting insulin (mIU/mL)×fasting glucose (mmol/L)]/22.5. Waist-to-height ratio (WHtR) was calculated as waist circumference divided by height (both in cm). Prediabetes was defined as fasting plasma glucose of 5.6 or greater and less than 7 mmol/L (20). T2DM was diagnosed on the basis of self-reporting or fasting plasma glucose of 7 mmol/L or greater.

### Antenatal Data and Fetal Growth Trajectories

Serial ultrasound measurements of abdominal circumference (AC), femur length (FL), and head circumference (HC) from 1333 mother-fetal pairs were used to develop fetal growth trajectories as described in detail previously (14). Briefly, linear regression models predicted SD scores for the 3 fetal anthropometric markers (AC, FL, and HC), adjusting for physiological factors influencing fetal growth (sex and ethnicity of the fetus, maternal height and parity). Seven AC, 5 FL, and 5 HC trajectory groups were identified (Supplementary Fig. S1) (21) by group-based trajectory modeling using a Stata plug-in (14).

### Statistical Analysis

#### Fetal growth trajectories and Gen2 adult measures of insulin resistance and adiposity

**Figure 1.**
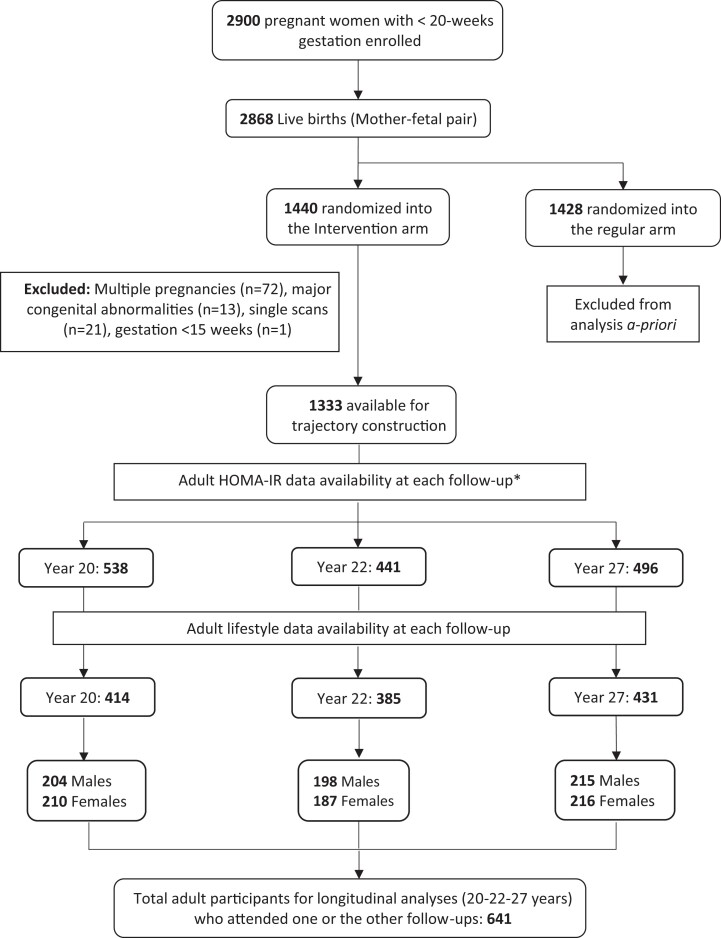
Representation of the original cohort and subsequent follow-up. HOMA-IR, Homeostatic Model Assessment of Insulin Resistance.

The relationship between fetal growth trajectory groups and adult HOMA-IR at 20, 22, and 27 years was examined using random coefficient mixed-effects linear regression. Bootstrapping with 500 replications minimized outlier influence and generated robust estimates. Data for trajectory groups and Gen2 adult lifestyle information were available for 414, 385, and 431 participants at 20, 22, and 27 years, respectively, representing 641 individuals who attended at least 1 follow-up. (Fig. 1). Statistical reasoning, scientific evidence, and availability of data were used to select the final set of confounders. Age at each follow-up, sex, ethnicity, alcohol intake, smoking, adult SES, educational status, and physical activity constituted the adult covariates. Maternal covariates included family income during pregnancy, smoking, alcohol drinking, weight gain, maternal BMI at 16 weeks, preterm pregnancy, gestational diabetes mellitus (GDM), family history of diabetes, uncomplicated HTN, complicated HTN, and breastfeeding. Family history of diabetes in the participants was examined using 2 variables: family history of diabetes in the grandparents of Gen2 (both maternal and paternal; self-reported by Gen1 parents; n = 164) and history of maternal diabetes in Gen1 before pregnancy (self-reported by mothers; n = 22). Log-transformation of HOMA-IR and insulin was performed because of evidence of significant skewing. Use of the mixed-modeling technique and bootstrapping accounted for missingness of data at one or more time points and for nonnormality. Selection of the most parsimonious model was based on a *P* value threshold, change in model estimates, and a priori knowledge of the risk factors. Mixed-model results were interpreted using a conservative approach based on both the global and local *P* value for the trajectory variable. SES, physical activity, and gestational age (for birth weight analyses) were normalized by *z* score standardization techniques. Model 1 examined the outcome variable (HOMA-IR) and trajectory groups adjusted for age, sex, female hormonal contraceptive use, ethnicity, SES, and adult lifestyle factors. Model 2 additionally adjusted for pregnancy covariates including uncomplicated hypertension during pregnancy, maternal alcohol drinking, and maternal BMI at 16 weeks. Both models 1 and 2 were adjusted for either BMI or WHtR (as a measure of adult adiposity) with results presented separately. BMI was centered at 25 and a second-order polynomial was used in some models. Sex-trajectory interaction was also examined. The relationship between birth weight as a continuous variable and HOMA-IR was examined using linear mixed modeling.

Results are presented as percentages, means with SDs, or medians with upper and lower quartiles. Stata v17.0 (Stata Corp) was used for all statistical analyses with 2-sided significance at *P* less than .05.

## Results

General characteristics of the Gen2 adults at 20, 22 and 27-years and their mothers (Gen1) during pregnancy are shown in Table 1. Ninety percent of Gen2 adults were White. Males had a higher BMI, waist circumference, and WHtR compared to females at all 3 ages. The highest proportion of central adiposity (identified by WHtR ≥0.5) was in women at 22 years and in men at 27 years. Median HOMA-IR values tended to be higher in females compared to males at 20 and 22 years. Alcohol was consumed by approximately 70% to 85% of participants at the 3 ages. The frequency of smoking was approximately 16% at 20 years and 21% to 24% at 27 years. Physical activity both in males and females was higher at 20 years than at 22 and 27 years. Female hormonal contraceptive use was 60% at 20 years and 49% at 27 years. Mean birth weight of the participants was between 3.30 and 3.42 kg. The distribution of Gen2 with prediabetes or diabetes was uniform across AC, FL, and HC trajectory groups at 20, 22, and 27-years (Supplementary Tables S1-S3) (21). T2DM was diagnosed in 1 person at 20 years and 5 each at 22 and 27 years.

**Table 1.** General characteristics of the participants (Gen2) at age 20, 22, and 27 years and their mothers (Gen1) during pregnancy

Characteristics	Y 20 (n = 414)		Y 22 (n = 385)		Y 27 (n = 431)	
	Males n = 204	Females n = 210	Males n = 198	Females n = 187	Males n = 215	Females n = 216
**Adult participants (Gen2)**						
Age, y Mean (SD)	20.1 (0.5)	20.0 (0.5)	22.2 (0.6)	22.1 (0.6)	26.8 (0.4)	26.8 (0.4)
BMI Median (Q1-Q3)	23.4 (21.2-26.4)	23.0 (20.9-25.8)	24.1 (22.0-27.5)	23.8 (21.4-28.8)	24.7 (22.6-27.6)	23.7 (21.3-27.4)
Waist circumference, cm Median (Q1-Q3)	79.1 (75.0-86.9)	74.0 (68.0-81.8)	83.3 (77.0-91.2)	76.4 (70.5-90.3)	85.6 (79.8-94.3)	76.6 (70.8-85.8)
Waist-to-Height ratio Median (Q1-Q3)	0.45 (0.42-0.49)	0.44 (0.41-0.49)	0.47 (0.43-0.51)	0.46 (0.42-0.54)	0.47 (0.44-0.52)	0.46 (0.42-0.52)
Waist-to-Height ratio ≥ 0.5, %	22.5	21.9	29.3	37.4	32.1	31.0
HOMA-IR Median (Q1-Q3)	0.48 (0.44-1.05)	0.57 (0.43-1.32)	1.50 (1.13-2.08)	1.75 (1.24-2.45)	1.13 (0.80-1.61)	1.12 (0.80-1.58)
Insulin, mIU/mL Median (Q1-Q3)	2.0 (2.0-4.4)	2.7 (2.0-5.8)	6.6 (5.0-9.0)	8.1 (6.0-11.2)	5.0 (4.0-7.0)	5.5 (4.0-8.0)
Glucose, mmol/L Mean (SD)	5.0 (0.4)	4.9 (0.4)	5.1 (0.4)	4.9 (0.4)	4.9 (0.4)	4.7 (0.6)
Alcohol drinkers, %	74.5	70.5	84.3	79.7	75.4	69.9
Alcohol intake, g/wk ethanol Median (Q1-Q3)	80.0 (0.0-200.0)	50.0 (0.0-110.0)	105.3 (36.8-238.0)	46.5 (11.1-106.7)	82.8 (10.0-203.3)	43.6 (0.0-120.7)
Smoking, % smokers	15.7	15.7	14.7	16.0	23.7	20.8
Physical activity, METmin/wk Median (Q1-Q3)	7386.0 (3672.0-12 834.0)	10441.5 (3291.0-17 280.0)	3822.0 (1866.0-7164.0)	2079.0 (810.0-4678.0)	2697.0 (1200.0-5112.0)	1972.5 (495.0-3582.0)
Socioeconomic status, SEIFA score Median (Q1-Q3)	1080.9 (1008.3-1118.4)	1069.7 (1006.8-1116.5)	1081.1 (1004.8-1119.6)	1066.5 (1004.7-1111.8)	1076.0 (997.1-1115.2)	1072.2 (1004.7-1117.8)
Educational status, %						
Category 1	76.5	73.8	52.3	51.7	26.1	24.5
Category 2	18.1	17.6	25.6	19.2	36.3	31.5
Category 3	5.4	8.6	22.1	29.1	37.7	44.0
*^a^* Contraceptive use in females, %	—	60.0	—	59.4	—	49.1
Ethnicity (% White)	86.7	90.0	88.9	89.8	90.7	91.2
Breastfed >6 mo, %	58.6	56.6	59.5	56.8	59.1	53.1
Birth weight, g Mean (SD)	3386.4 (517.9)	3304.9 (491.4)	3366.3 (544.6)	3303.2 (528.1)	3413.7 (575.9)	3337.6 (531.2)
**Maternal (Gen1)**						
BMI at 16 wk Median (Q1-Q3)	22.8 (20.8-24.7)	23.1 (21.1-25.8)	22.7 (20.7-24.4)	23.4 (21.1-26.2)	22.9 (21.1-25.3)	23.4 (21.1-25.9)
Preterm delivery, %	4.4	5.2	5.1	5.4	6.1	4.2
GDM, %	2.5	1.4	3.5	1.6	3.3	0
Maternal smoking, %	20.6	23.3	18.2	24.1	16.7	25.5
Alcohol drinkers, %	60.8	56.7	58.6	54.6	58.6	57.9
Uncomplicated HTN, %	27.7	20.0	24.9	20.9	26.2	19.4
Complicated HTN, %	3.0	2.9	2.5	1.1	3.3	2.3
Low income, %	31.7	34.6	36.9	34.3	38.4	34.0

Educational status: category 1, those completing high school; category 2, those with apprenticeship or vocational training; category 3, those in university. Low income, family income in 1989 to 1991, low being annual income less than $24 000 (AUS).

Abbreviations: BMI, body mass index; Complicated HTN, HTN during pregnancy with proteinuria (>2 + on dipstix test) or 300 mg on 24-hour urinary protein excretion or preterm delivery; GDM, gestational diabetes mellitus; HOMA-IR, Homeostatic Model Assessment of Insulin Resistance; hs-CRP, high-sensitivity C-reactive protein; HTN, hypertension; SEIFA, socioeconomic indexes for areas; Q1, first quartile or 25th percentile, Q3, third quartile or 75th percentile; Uncomplicated HTN, history of HTN before pregnancy or HTN during pregnancy without proteinuria or preterm delivery.

Contraception refers to the female use of hormonal contraceptives.

In relation to maternal characteristics (Gen1), up to 6% reported preterm deliveries, 3.5% gestational diabetes, 27.7% uncomplicated HTN and 3.3% complicated-HTN (see Table 1). Up to 25% of Gen1 mothers smoked and 60% consumed alcohol during pregnancy. About 32% to 38% of Gen1 families had an annual income of less than $24 000 (AUS) at the time of pregnancy.

### Growth Trajectories and Gen2 Homeostatic Model Assessment of Insulin Resistance

#### Relationship of abdominal circumference trajectories with Homeostatic Model Assessment of Insulin Resistance

**Figure 2.**
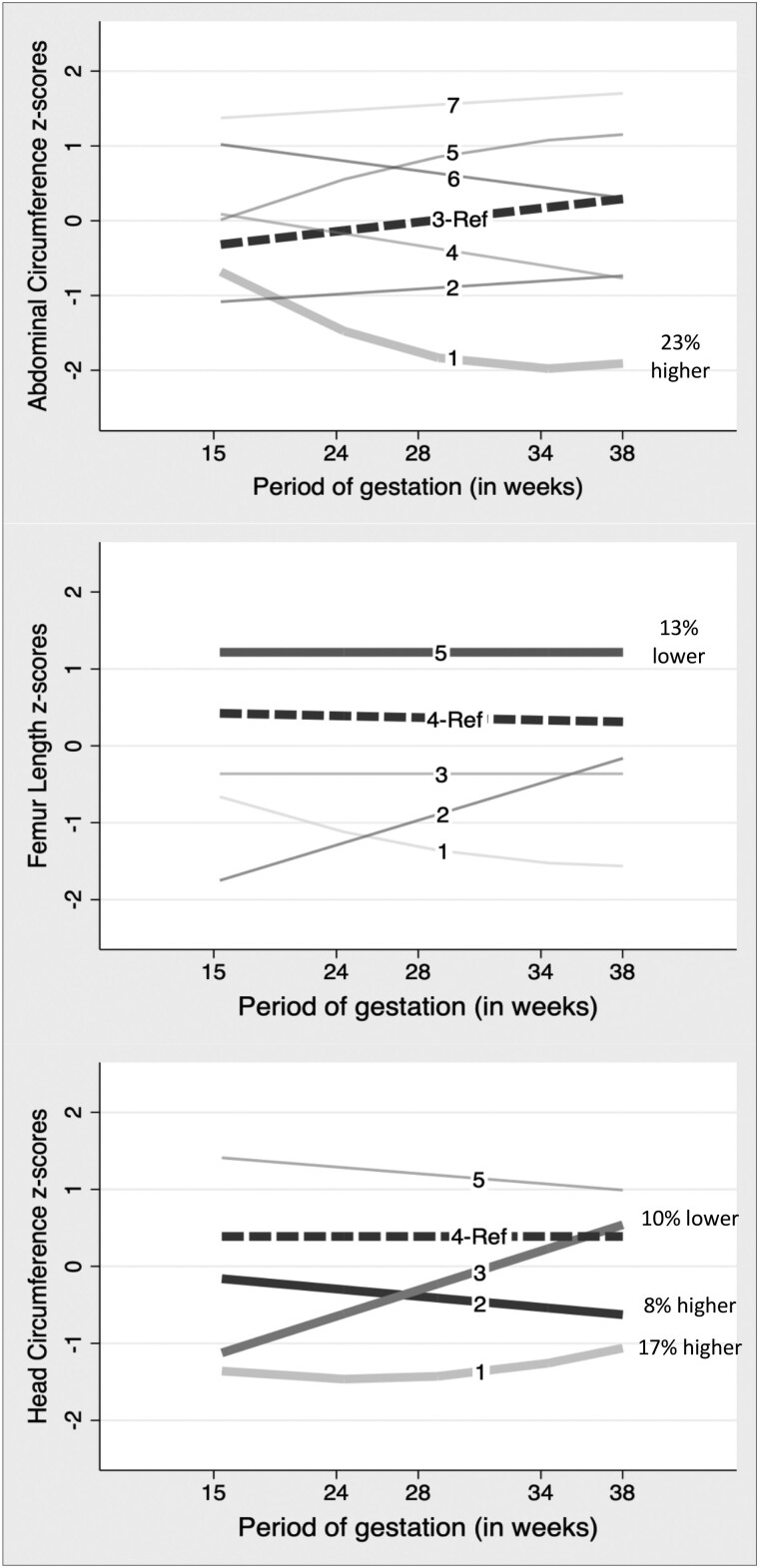
Fetal growth trajectories highlighting the association with adult HOMA-IR. Y-axis: SD scores or *z* scores for abdominal circumference (AC), femur length (FL), and head circumference (HC). X-axis: gestational age in weeks. Dashed lines (▬ ▬) represent the reference trajectory group while solid-bold lines (▬) represent those trajectory groups having a significant association with reference group. The percentages represent change in HOMA-IR values in the respective trajectory groups, compared to the HOMA-IR value in the reference group. HOMA-IR, Homeostatic Model Assessment of Insulin Resistance.

Participants in group 1 (low-falling) had 26% higher HOMA-IR (β = 1.26; *P* = .005), compared with the reference group (group 3), after adjusting for age, sex, hormonal contraceptive use, ethnicity, SES, BMI, and alcohol intake (model 1: see Table 2, Fig. 2, and Supplementary Table 4) (21). The association remained significant with further adjustment for maternal covariates (model 2: see Table 2 and Supplementary Table S4) (21). Maternal alcohol drinking in pregnancy (model 2) was found to be independently associated with HOMA-IR (*P* = .004).

**Table 2.** Fetal growth trajectories and their association with Homeostatic Model Assessment of Insulin Resistance

AC							
*^a^* Trajectories (Group membership)	Group 1 Low-falling 4.9% (n = 28)	Group 2 Low-stable 13.0% (n = 75)	Group 4 Average-falling 21.2% (n = 122)	Group 5 Average-rising 13.0% (n = 75)	Group 6 High-falling 17.2% (n = 99)	Group 7 High-rising 5.6% (n = 32)	Global *P*
HOMA-IR (using BMI) exponentiated β coefficients							
Model 1 Model 2	1.26*^c^*	1.01	1.03	0.96	1.06	0.88	.011
	1.27*^c^*	1.01	1.03	0.96	1.07	0.90	.014
HOMA-IR (using WHtR) exponentiated β coefficients							
Model 1 Model 2	1.23*^c^*	1.01	1.00	0.96	1.04	0.89*^b^*	.010
	1.23*^c^*	1.01	1.00	0.96	1.05	0.91	.015

FL					
*^a^* Trajectories (Group membership)	Group 1 Low-falling 5.6% (n = 32)	Group 2 Very low-rising 8.0% (n = 46)	Group 3 Low-stable 30.8% (n = 177)	Group 5 High-stable 12.7% (n = 73)	Global *P*
HOMA-IR (using BMI) exponentiated β coefficients					
Model 1 Model 2	0.99	0.94	0.98	0.88*^c^*	.026
	0.99	0.94	0.98	0.87*^c^*	.019
HOMA-IR (using WHtR) exponentiated β coefficients					
Model 1 Model 2	0.99	0.96	0.99	0.87*^d^*	.005
	0.99	0.96	0.99	0.87*^d^*	.005

HC					
*^a^* Trajectories (Group membership)	Group 1 Low-stable 7.8% (n = 45)	Group 2 Average-falling 30.1% (n = 173)	Group 3 Low-rising 8.2% (n = 47)	Group 5 High-stable 12.2% (n = 70)	Global *P*
HOMA-IR (using BMI) exponentiated β coefficients					
Model 1 Model 2	1.20*^c^*	1.08*^b^*	0.91*^c^*	0.92	<.001
	1.20*^c^*	1.08*^b^*	0.90*^b^*	0.92	<.001
HOMA-IR (using WHtR) exponentiated β coefficients					
Model 1 Model 2	1.17*^b^*	1.07*^b^*	0.90*^b^*	0.91*^b^*	<.001
	1.17*^b^*	1.08*^b^*	0.90*^b^*	0.92	<.001

Linear mixed modeling results displayed. Sample size: model 1 and 2: 1147 observations representing 575 participants who attended one or more follow-ups. Covariates: model 1, age, sex, female contraceptive use, ethnicity, socioeconomic status, adult BMI, and alcohol intake; model 2, model 1 plus maternal alcohol drinking.

Abbreviations: AC, abdominal circumference; BMI, body mass index; FL, femur length; HC, head circumference; HOMA-IR, Homeostatic Model Assessment of Insulin Resistance; WHtR, waist-to-height ratio.

Trajectories based on 1333 participants; reference group is group 3 (average-stable) with a membership of 25.0% (n = 144) for AC, group 4 (average-stable) with a membership of 43.0% (n = 247) for FL, and group 4 (average-stable) with a membership of 41.7% (n = 240) for HC.

Statistically significant *P* values: *^b^*less than .05, *^c^*less than .01, *^d^*less than .001.

A sex-trajectory interaction was detected (*P* = .025) and in sex-specific analysis, males in group 7 (high-rising) had 22% lower (β = .78; *P* = .008) HOMA-IR (adjusted for age, BMI, ethnicity, and SES), while females in group 1 (low-falling) had 56% higher (β = 1.56; *P* < .001) HOMA-IR (adjusted for age, contraceptive use, BMI, ethnicity, and SES) (model 1: Supplementary Table S5) (21). These associations remained statistically significant with additional adjustment for maternal covariates (model 2: Supplementary Table S5) (21).

Using WHtR to adjust for adult adiposity instead of BMI, group 1 participants were associated with 23% higher HOMA-IR (*P* = .008), while group 7 participants were associated with 11% lower HOMA-IR (*P* = .040), compared to the reference group (model 1: see Table 2 and model-1: Supplementary Table S6) (21).

#### Relationship of femur length trajectories with Homeostatic Model Assessment of Insulin Resistance

Compared with reference group 4, participants in group 5 (high-stable) were associated with 12% lower HOMA-IR (β = .88; *P* = .002) (model 1: see Table 2, Fig. 2, and Supplementary Table S7) (21). This association persisted with adjustment for maternal covariates (model 2: see Table 2 and Supplementary Table S7) (21) and showed maternal alcohol drinking was independently associated with HOMA-IR (*P* = .002). There was no sex-trajectory interaction (*P* = .812). Using WHtR instead of BMI produced similar β coefficients for group 5 (Supplementary Table 8) (21).

#### Relationship of head circumference trajectories with Homeostatic Model Assessment of Insulin Resistance

Participants in group 1 (low-stable) and group 2 (average-falling) were associated with 20% (β = 1.20; *P* = .006) and 8% (β = 1.08; *P* = .021) higher HOMA-IR, respectively, compared to reference group 4 (average-stable) (model 1: see Table 2, Fig. 2, and Supplementary Table S9) (21). In the same model, participants in group 3 (low-rising) were associated with 9% (β = .91; *P* = .021) lower HOMA-IR compared to the reference group. Adjustment for maternal covariates (model 2) resulted in similar coefficients, with maternal alcohol drinking independently associated with HOMA-IR (*P* = .002). There was no sex-trajectory interaction (*P* = .639).

Replacing BMI with WHtR for adiposity as a covariate resulted in similar coefficients for groups 1, 2, and 3 (see Table 2 and Supplementary Table S10) (21). Additionally, with WHtR as a covariate, group 5 (high-stable) participants were associated with 9% (β = .91; *P* = .042) lower HOMA-IR compared to the reference group (model 1: see Table 2 and model 1: see Supplementary Table S10) (21).

In all analyses examining associations between AC, FL, and HC trajectories and HOMA-IR, family history of diabetes was not statistically significant in the final model and did not significantly change the coefficients.

### Relationship of Growth Trajectories With Insulin and Glucose

Associations between growth trajectories and insulin were similar to HOMA-IR (Supplementary Table S11) (21). However, the 2 falling AC trajectory groups: group 1 (low-falling) and group 6 (high-falling) had higher fasting glucose (β = .10; *P* = .014 and β = .09; *P* = .047, respectively), compared to reference group 3. No association was found for FL and HC trajectories and glucose (Supplementary Table S11) (21).

### Relationship of Birthweight and Adult Homeostatic Model Assessment of Insulin Resistance and Waist-to-Hip Ratio

Birth weight (kg), adjusted for gestational age, was inversely associated with HOMA-IR (β = .86; *P* < .001), representing 14% lower HOMA-IR for every 1-kg increase in birth weight, in models adjusted for age, sex, female contraceptive use, ethnicity, SES, BMI, and alcohol intake (model 1: Supplementary Table S12) (21). This association remained statistically significant with adjustment for maternal covariates (model 2).

## Discussion

This study has shown significant relationships between in utero fetal growth trajectories from early in pregnancy and measures of insulin resistance in the offspring during adulthood. In particular, trajectories reflecting lower-than-average HC and AC, representing restricted fetal growth, were associated with higher adult HOMA-IR, while the trajectory reflecting above-average and increasing abdominal growth were associated with lower HOMA-IR. Similarly, above-average growth in FL and HC, and accelerated growth in HC, were associated with lower HOMA-IR. The associations were independent of maternal and adult lifestyle factors and showed some differences between the sexes. For example, the relation between the restricted abdominal growth patterns and HOMA-IR was substantially greater in females than males. The results complement our previous findings of relationships between fetal growth patterns and markers of adult risk factors for cardiometabolic diseases including blood pressure, BMI, waist circumference, and hs-CRP (14, 15). The associations between HOMA-IR and growth trajectories in this study were mainly driven by serum insulin. Use of WHtR instead of BMI as a covariate for the relationship between trajectories and HOMA-IR showed similar results, although in some instances, it strengthened the coefficients, which highlights the value of WHtR as a measure for assessing cardiometabolic risk (22, 23).

Fetal growth is determined by many interactions including those between genetics, environmental factors, placental nutrition, and oxygen, and is an identified risk factor for the development of several diseases in adulthood including T2DM (6, 10, 24, 25). Poor maternal nutrition during gestation and consequent placental insufficiency can cause lower-than-average growth of fetal HC, reflecting diminished brain size and poor development (26). The development of the hypothalamic-pituitary-adrenal axis, responsible for neuroendocrine regulation of insulin metabolism and immune responses, begins in early fetal life. One could speculate that reduced head size and related in utero environmental stressors during critical periods of growth can alter normal neuropeptide synthesis, thereby disrupting proper functioning of the hypothalamic-pituitary-adrenal axis and leading to a dysregulation of glucose-insulin homeostasis (27). AC, on the other hand, approximates growth of fetal liver and abdominal subcutaneous fat. Restricted fetal abdominal growth could cause reduced β-cell mass with reduced secretory capacity in the pancreas, reduced glucose uptake, and increased gluconeogenesis in the liver, increased lipid oxidation in the muscles, and decreased insulin inhibition of lipolysis in the adipocytes, all cumulatively leading to glucose intolerance (28). While these adaptive mechanisms help in fetal survival, many persist into postnatal life and may increase the propensity for future abdominal obesity, insulin resistance, and T2DM. Preterm birth, exposure to antenatal corticosteroids in growth-restricted fetuses, and rapid catch-up growth in infancy can also contribute to the risk of visceral adiposity and insulin resistance in later life (29, 30). The role of insulin in mediating normal growth in healthy individuals and promoting adiposity during hyperinsulinemia through maintenance of the insulin–growth hormone (GH)–insulin-like growth factor 1 (IGF-1) axis is well recognized (31). However, the Raine Study does not have measures of insulin, GH, or IGF-1 of the mothers during pregnancy and GH or IGF-1 of the offspring during childhood, adolescence, and adulthood.

Lifestyle maternal characteristics during pregnancy have been identified as determinants of the long-term health of the offspring (32). As reported previously, mothers with a fetus in a restricted-growth trajectory in this study cohort were more likely to experience a preterm delivery. Similarly, mothers of a fetus with restricted abdominal growth had a higher BMI at 16 weeks of gestation, while those with a fetus with restricted head growth were more likely to smoke during pregnancy (14). Our findings reinforce the importance of public health strategies targeting lifestyle interventions like maternal obesity and smoking to ameliorate the future risk of diabetes in the offspring.

Fetal growth during pregnancy has largely been assessed using birth weight and less commonly using ultrasound-based, in utero anthropometric growth markers (13). Systematic reviews and meta-analyses have shown an inverse relationship between birth weight and measures of insulin resistance and/or T2DM during adolescence and adulthood (33-36). In our analysis, HOMA-IR in adulthood was 14% lower for every 1-kg increase in birth weight, after adjusting for adult lifestyle and maternal factors. As we have previously shown, birth weight may not provide an accurate measure of different patterns of fetal development in utero (14). Use of growth trajectories as a measure of fetal growth therefore provides a more complete picture of the dynamics of the intrauterine environment that may be contributing to the programming of adult diseases (25, 37).

Our analyses have also accounted for maternal factors influencing fetal growth such as maternal drinking, maternal BMI, and HTN in pregnancy, as well as family history of diabetes. In particular, maternal alcohol drinking was found to be independently associated with HOMA-IR. Several animal studies have reported strong evidence of linkage between prenatal alcohol exposure and T2DM through alteration of insulin expression and IGF signaling leading to metabolic dysregulation in the offspring (38, 39). However, establishing causality in humans is an ethical challenge and alcohol consumption during pregnancy continues to be major health concern with an estimated global prevalence of about 10% (40). About 58% mothers in the Raine Study consumed alcohol in pregnancy, possibly because of a lack of awareness about the detrimental effects of fetal alcohol exposure during 1989 to 1991. The HAPO study on associations with maternal BMI showed that higher maternal BMI is strongly associated with excess fetal growth and adiposity (41). We have previously shown strong associations of maternal BMI with offspring adiposity (15) in the Raine cohort. In the present study, although maternal BMI was significantly associated (*P* < .001) with offspring HOMA-IR in the univariate analysis, no statistically significant association was found in the final multivariable analysis, most likely as the latter was adjusted for offspring adiposity.

In this study, we showed significant relationships between fetal growth patterns and HOMA-IR, adjusting for either BMI or WHtR as a measure of adult central adiposity. Both BMI and WHtR were found to be independent significant predictors of diabetes risk in adulthood. Jayedi et al (42) have recently shown significant associations between different anthropometry measures and the risk of T2DM in the general population. An increase in WHtR by 0.01 units, BMI by 1 unit, and waist circumference by 1 cm are associated with a 7.3%, 14.4%, and 6.1%, respectively, higher risk of T2DM (42). A meta-analysis by Ashwell et al (23) provides robust evidence from studies involving more than 300 000 adults from several ethnic groups, showing that WHtR is a better measure than BMI and waist-circumference in predicting cardiometabolic risk. WHtR has also been recognized as a more reliable measure to distinguish differences in body fat distribution, thereby allowing more accurate interpretations of visceral adiposity both in men and women (43). Use of WHtR in the present analysis strengthens our previous findings that showed significant associations between trajectories and adult BMI and waist circumference and builds strong evidence of association between early fetal growth and risk factors for T2DM in adulthood.

Strengths of this study include the application of serial ultrasound measures to identify unique fetal growth patterns and longitudinal analysis of adult measures across 3 ages from 20 to 27 years. The Raine Study cohort is a well-characterized, homogeneous population of primarily White individuals of above-average SES. The cohort is representative of the contemporary Western Australian population both at the time of recruitment and at subsequent follow-ups, even allowing for attrition (16, 44, 45). Information related to pregnancy, birth, and follow-up visits has been carefully documented through medical records and online databases, eliminating any concerns for recall bias. Longitudinal analysis using mixed modeling made efficient use of the available data and allowed us to examine relationships independent of a number of adult and maternal factors. Study limitations include the relatively small sample for some trajectories, restricting the use of multiple comparisons to explore sex-trajectory interactions and limiting our statistical ability to detect more subtle relationships in subgroup analyses. Nonavailability of sibling rank data was another limitation, but we did account for mother's parity during trajectory modeling. Low rates of GDM in this cohort could be due to a lack of routine testing in late pregnancy and lower rates of maternal obesity in the 1990s. Allocation of HTN pregnancies was performed by midwives based on the retrieval of medical records, which could have led to an overestimation of the proportion of pregnancies with uncomplicated HTN. Additionally, Gen1 mothers were recruited from King Edward Memorial Hospital, Perth, which historically has been associated with a higher proportion of pregnancies with complications (38.6% compared to 30% in the contemporary Western Australian obstetric population) (44). However, follow-up studies have shown that those who attended subsequent follow-ups were more representative of the contemporary population. Importantly, we adjusted for uncomplicated HTN in our analysis. While analyses accounted for several antenatal, postnatal, current lifestyle, and socioeconomic factors, we cannot exclude the contribution of unmeasured residual confounding factors, including a possible genetic influence on the relationship between in utero growth trajectories and adult insulin sensitivity. Last, assumptions about causality need a cautious approach because of the observational nature of the study.

To our knowledge, this is the first study to investigate the relationship between fetal growth trajectories based on serial ultrasound and markers of insulin resistance in adulthood using novel modeling techniques. While the Raine Study cohort is relatively young with a low incidence of diabetes, follow-up is ongoing and will provide more evidence as the cohort matures. A global increase in the rates of obesity in the 30-year period since the Raine Study was initiated is likely to further amplify the metabolic risk shown in our study. Together with our previous results, these findings provide further insight into our understanding of early gestational determinants of adult diabetes. Early identification of fetuses with future risk based on ultrasound-based growth assessment could play an important role in long-term risk-prevention of diabetes and related cardiometabolic diseases.

## Data Availability

The data sets generated during and/or analyzed during the present study are not available. The Raine Study is committed to a high level of confidentiality of the data in line with the informed consent provided by participants. Requests for data should be directed to the Raine Study Executive.

## Abbreviations

AC: abdominal circumference
BMI: body mass index
FL: femur length
GDM: gestational diabetes mellitus
GH: growth hormone
HC: head circumference
HOMA-IR: Homeostatic Model Assessment of Insulin Resistance
hs-CRP: high-sensitivity C-reactive protein
HTN: hypertension
IGF-1: insulin-like growth factor 1
MET: metabolic equivalent
SEIFA: socioeconomic indexes for areas
SES: socioeconomic status
T2DM: type 2 diabetes mellitus
WHtR: waist-to-height ratio
